# Childhood maltreatment and psychopathy in Chinese juvenile offenders: person-centered perspective

**DOI:** 10.1186/s40359-024-01634-8

**Published:** 2024-03-08

**Authors:** Yuanhua Yang, Jie Yu, Suxian Zhang, Qing Xie

**Affiliations:** 1 Department of Management, Hunan Police Academy, 410138 Changsha, Hunan China; 2 Mental Health Education and Consulting Center, Hunan Normal University, 36 Lushan Road, 410081 Changsha, Hunan China

**Keywords:** Childhood maltreatment, Latent Profile Analysis, Chinese juvenile offenders, Psychopathy

## Abstract

**Background:**

Childhood maltreatment stands as a salient risk factor in the etiology of juvenile delinquency, with a profound impact on the behavioral trajectories of young offenders. However, there is limited research on latent profile analysis to explore distinctive patterns of childhood maltreatment in Chinese juvenile offenders. Consequently, there is a lack of understanding regarding the associations between maltreatment profiles and relevant variables in this context. The present study aimed to explore meaningful subgroups of childhood maltreatment in juvenile offenders, and we further examined the associations between subgroups and multiple outcomes especially psychopathy.

**Methods:**

The data was obtained from a sample of Chinese juvenile offenders (*N* = 625, *M* age = 17.22, *SD* = 1.23). This study employed a latent profile analysis (LPA) based on factor scores of the Childhood Trauma Questionnaire-Short Form to identify the subgroups and examined the differences across subgroups using outcomes variables including psychopathy, callous-unemotional traits, aggression and anxiety. This study includes three self-report measures to evaluate psychopathy, with due regard for the nuanced considerations on the factor structure inherent in the conceptualization of psychopathy.

**Results:**

Two subgroups were identified, including the non-maltreatment subgroup (80.2%) and the maltreatment subgroup (19.8%). Maltreatment subgroup was characterized by a greater level of all types of maltreatment with particularly higher of emotion neglect. Besides, we found that maltreatment subgroup showed a significantly higher level of psychopathy across multiple self-report measures, and greater callous-unemotional traits, lack of empathy, aggression and anxiety. We found two subgroups of child maltreatment in Chinese juvenile offenders.

**Conclusions:**

These findings may provide a further understanding of childhood maltreatment and the clinical intervention on psychopathy in the early period.

## Introduction

Childhood maltreatment (CM) is considered as a global public health concern across the world. Childhood maltreatment is a multidimensional concept that describes abuse and neglect occurring to children under the age of 18, including physical abuse, physical neglect, emotional abuse, emotional neglect, and sexual abuse [1]. Children and adolescents who experienced abused or neglected have a significantly increased risk of developing a variety of psychosocial development and mental health as adults [2], including internalizing behaviors [3] and externalizing behaviors, such as aggressive behaviors [4] and substance abuse [5]. For instance, children with maltreatment were more aggressive [4]. The physical abuse had associations with externalizing behaviors, delinquency, and drugs use [6]. Physical neglect refers to the failure of caregivers to meet a child’s fundamental physical needs, such as food, shelter, clothing, safety, and health care. Emotional neglect is characterized by the failure of caregivers to fulfill the fundamental emotional and psychological requirements of children, encompassing the provision of love, a sense of belonging, nurturance, and support. Neglect during childhood can give rise to tangible or conceivable harm concerning the child’s health, survival, development, or dignity within a relationship characterized by responsibility, trust, or power dynamics [3]. Emotional abuse manifests through verbal assaults that undermine a child’s sense of worth or overall well-being. This category encompasses behaviors such as insults and expressions of disdain directed explicitly at the child. The impact of emotional abuse can be profound and may result in long-term mental consequences, including anxiety, depression, and low self-esteem [3]. Sexual abuse pertains to any sexual contact or conduct involving a person under the age of 18 and an adult or older individual, which may lead to some consequences such as trauma, self-harm, difficulty forming relationships [7].

Numerous studies indicated that a significant prevalence of child maltreatment within Chinese households [8–9] and previous evidence has indicated instances of child neglect specifically within single-child families in China [10]. Therefore, it is important to understand childhood maltreatment to target the interventions and implications. However, it remains unclear the characteristics of childhood maltreatment profiles. Thus, this study aimed to explore childhood maltreatment profiles and validate the profiles with relevant variables such as psychopathy.

### Context of the study

Childhood maltreatment has been shown to increase the risk of later violence perpetration from a meta-analytical study [11]. Also, the experiences of childhood maltreatment are common for youth involved in juvenile offenders [12]. Incarcerated juveniles experienced histories of trauma [12] and the majority of juvenile offenders reported a history of at least one traumatic experience [12]. For example, Abram and colleagues [13] reported that detained juvenile offenders commonly reported experiences of child maltreatment, such as emotional, physical, and sexual abuse. Moreover, multiple maltreated youths, when compared to non-maltreated counterparts or those experiencing a single type of abuse, face an elevated risk of engaging in self-reported delinquent behaviors during adolescence [14]. Additionally, they are more likely to report criminal activities in adulthood [15]. Besides, previous findings suggested that childhood maltreatment may be considered as a valid predictor for reoffending in male juvenile offenders [16].

Over the past decade, there has been a growing trend in the field of maltreatment research toward the adoption of person-centered approach [17]. Historically, the studies in the literature have commonly involved a focus on single type of maltreatment or rely on classification based on the presence of any type of maltreatment. However, it is necessary to move beyond a focus on type and begin to approach maltreatment as a multidimensional construct that can be conceptualized across several dimensions to fully understand the causes and consequences of maltreatment [18]. The person-centered approach holds significant promise for research seeking to comprehensively capture the nature of maltreatment in this context. Latent profile analysis (LPA) is a person-centered approach to identify subgroups or profiles using continuous indicators and allows for distinct patterns among potential indicators [19]. Based on the characteristics of different subgroups/profiles, it can provide an alternative understanding for clinical implications and treatment strategy for specific patterns. In recent years, an increasing number of researchers have recommended to employ the LPA to identify meaningful subgroups of child maltreatment [20–22]. According to the findings from a meta-analysis about latent classes of maltreatment [23], the LPA studies published before 2016 mainly focused on the community samples such as child samples and combined age samples (0–29 years) in Western countries. For example, Pears et al. reported that four latent classes were found in a sample 117 preschool-aged foster children with maltreatment reports [24]. Besides, Romano and colleagues [25] found there were two profiles in a sample of at-risk pregnant adolescents and emerging adults (*N* = 252) from Canada, including low/no abuse (79%) and physical, sexual, emotional, and emotional neglect (21%). Among Chinese samples, two (no maltreatment vs. multiple maltreatment) or four profiles (psychological non-support, low-maltreated, high-maltreated, and severe-maltreated) were recently found in community child and adolescents [22, 26]. Furthermore, Lin and colleagues identified three profiles of child maltreatment among children with oppositional defiant disorder [27].

In the incarcerated samples, the latent class of childhood maltreatment has been uncertain. Aebi found three subgroups (Low/no abuse; 74%, Physical and emotional; 18%, Physical, sexual, and emotional; 8%) in a sample of male adolescent and emerging adult juvenile offenders in Vienna, Austria [28]. Debowska and Boduszek identified three subgroups (low abuse, high physical and emotional abuse, poly-victimized) in a large sample of incarcerated males in the Republic of Poland [21]. In China, Zhang and Zheng reported four profiles including minimal maltreatment (61.5%), low abuse and high neglect (26.6%), high sexual abuse with multiple maltreatment (4%), and high physical and emotional maltreatment (7.8%) in adult male offenders [29]. It seems that there were three to four profiles of maltreatment in the offenders. Thus, more studies need to further investigate the maltreatment profiles in the incarcerated sample especially in the early period.

Previous studies suggested that there was a link between childhood maltreatment and psychopathy [30]. Psychopathy is a personality disorder characterized by multidimensional facets, including affective (e.g., callous/lack of empathy), interpersonal (e.g., grandiosity and lying), behavioral instability (e.g., impulsivity, poor behavioral controls) [31]. Early childhood maltreatment has been considered as a predictor for the development of psychopathy. According to the findings from a meta-analysis of the relationship between childhood maltreatment and psychopathic traits [30], it suggested that there was a moderate link between overall childhood maltreatment and psychopathy. The majority of the studies in psychopathic offenders retrospectively reported more childhood abuse than nonpsychopathic offenders. However, these findings were mostly based on the variable-centered approach and thus the associations between maltreatment profiles and psychopathy remains unclear. Also, it has been unclear whether the association between maltreatment and psychopathy remain stable across different measures given there were some inconsistencies on the factor structure of psychopathy with multiple assessment tools [32].

In terms of affective facet that was considered as main feature in youth called callous-unemotional (CU) traits, it has also been uncertain in the relationship with childhood maltreatment profiles in Chinese juvenile offenders. A study examined maltreatment profiles among incarcerated boys with CU traits in the United States [33] and implied that poor emotional experiences provided by neglectful surroundings may lead to one of developmental pathways to CU traits in youth [34]. Few studies explored the association between maltreatment profiles and CU traits in China especially in offenders. Therefore, the findings in Western were unknown to generalize to the juvenile offenders in China especially with consideration of the differences of emotional expression from two cultural backgrounds.

### The current study

The present study aimed to investigate the profiles of childhood maltreatment in a sample of Chinese juvenile offenders using latent profile analysis. Furthermore, we also examined the differences between maltreatment profiles on multiple outcome variables to validate the profiles of maltreatment, including psychopathy, callous-unemotional traits, anxiety, aggression and empathy. With consideration of the inconsistency on the number of factor structure of psychopathy [31, 35], the current study intended to used three self-report measures for assessing psychopathy, including Antisocial Process Screening Device–Self-Report Version (APSD-SR), Proposed Specifiers for Conduct Disorder scale (PSCD) and Youth Psychopathic Traits Inventory (YPI) and examined whether similar findings would be found.

Based on the previous findings among adolescents and offenders [22, 36], we expected that there would be significant differences between maltreatment profiles and outcome variables. In particular, it was expected that the profile with the higher maltreatment level would score higher on psychopathy, callous unemotional traits, anxiety and aggression and lower empathy.

The findings of this study may provide significant implications to understand the characteristics of maltreatment profile and the associations with outcomes variables during early developmental stage in justice settings. From a clinical perspective, optimizing intervention strategies would be advantageous for practitioners by tailoring their approaches with greater precision to the distinct forms of maltreatment experienced by individuals.

## Method

### Participants

The sample of 625 juvenile offenders (*M* = 17.22, *SD* = 1.23) was recruited from one juvenile detention center managed by Guangdong Prison Administrative Bureau in Guangzhou, the capital and largest city of Guangdong Province in southern China. Within the framework of the Chinese criminal justice system, a juvenile offender is defined as an individual aged between 14 and 18 years who has committed a criminal offense. Attainment of full criminal responsibility occurs at the age of 16, while those aged 14 or older but below 16 bear criminal responsibility solely in instances involving intentional homicide, intentional injury, death, rape, robbery, drug trafficking, arson, explosion, or poisoning. Notably, individuals convicted of crimes between the ages of 14 and 18 are subject to lighter, mitigated penalties relative to their adult counterparts.

Although a subset of participants (8%) surpassed the age threshold of 18, their initial entanglement with the criminal justice system transpired during their adolescence, and they presently remain detained within the juvenile detention center. Predominantly, the detentions of these individuals stemmed from the commission of severe offenses, such as robbery and assaults.

Participants were predominantly Han ethnicity (87.0%) and 13.0% other ethnic minority. The majority (77.3%) was from nuclear families while the others lived with single parents or in divorced families. More than a half of samples lived with their parents under the age of 12 (66.9%), 27.7% with their grandparents and 5.4% with relatives. Regarding parents’ educational backgrounds, most were at the junior middle school (*N* = 290; 46.4% for fathers and *N* = 356; 57.0% for mothers) and high school educational level (*N* = 258; 41.3% for fathers and *N* = 214; 34.2% for mothers) and the others were at the primary school (0.2% for fathers and 0.3% for mothers) and had a bachelor degree or greater educational level (*N* = 76; 12.2% for fathers and *N* = 53; 8.5% for mothers).

### Measures

#### Childhood trauma questionnaire-short form (CTQ-SF)

The original CTQ-SF is the most commonly self-reported screening questionnaire to assess childhood trauma and abuse experiences in both clinical and non-referred groups developed by Bernstein et al. [1]. It included 28 items (25 clinical items and 3 validity items) with five subscales, including Emotional abuse, Physical abuse, Sexual abuse, Emotional neglect, and Physical neglect. Each item is measured on a 5-point Likert scale ranging from 1 (never true) to 5 (very often) and higher scores indicate more severe trauma exposure. The minimization/denial scale was used to screen for the likelihood of underreporting trauma experience with three questions (Item 10, 16, and 22). The scale was translated into the Chinese version and had acceptable psychometric properties in Chinese adolescents [37]. In the current study, the models fit indices were acceptable (RMSEA = 0.05, CFI = 0.85; SRMR = 0.06). The Cronbach’s alpha coefficients for the five subscales (emotional abuse, physical abuse, emotional neglect, sexual abuse, physical neglect subscale) and total score were 0.47, 0.53, 0.79, 0.76, 0.69, and 0.70, respectively.

#### Antisocial process screening device–self-report version (APSD-SR)

The APSD-SR is a self-report scale to assess psychopathic traits in youth [31]. It included 20 items with three subscales, including Callous-unemotional traits (6 items), Narcissism (7 items) and Impulsivity (5 items). Two items (Item 2 and Item 6) were only used to calculate the total scores. Each item is rated on a 3-point Likert scale ranging from 0 (not at all true) to 2 (definitely true) and higher scores indicate higher levels of psychopathic traits. Prior studies have demonstrated acceptable reliability and validity of the APSD in Chinese samples [38]. In the current sample, the Cronbach’s α coefficient for the total score was 0.72.

#### Proposed specifiers for conduct disorder scale (PSCD)

The PSCD is a 24-item questionnaire to measure psychopathic traits in youth. It consists of four subscales with six items in each subscale, including grandiose-manipulative traits, Callous-unemotional traits, Daring-impulsive traits, and Conduct disorder [39]. Each item is rated on a 3-point Likert scale ranging from 0 (not at all true) to 2 (definitely true). Higher scores indicate greater psychopathic traits. The Chinese version has shown acceptable psychometric properties in children and adolescents [40]. In the current study, the Cronbach’s α coefficient for the total score was 0.81.

#### Youth psychopathic traits inventory (YPI)

The YPI is a self-report measure to assess psychopathic traits for youth samples [35]. This scale included 50 items with 10 subscales (i.e., Dishonest charm, Grandiosity, Lying, Manipulation, Remorselessness, Unemotionality, Callousness, Thrill-seeking, Impulsiveness and Irresponsibility). Each item is rated on a 4-point Likert scale ranging from 0 (does not apply at all) to 4 (applies very well). The Chinese version had a satisfactory reliability and validity in Chinese youth [38]. In the current study, the Cronbach’s α coefficient for the total score was 0.90.

#### Inventory of callous-unemotional traits (ICU)

The ICU is one of the most widely used scale to assess CU traits in youths [41]. It is a 24-item multi-informant rating measure with three subscales, including Callousness (11 items), Uncaring (8 items), and Unemotionality (5 items). Each item is measured on a 4-point Likert scale ranging from 0 (does not apply at all) to 4 (applies very well). Higher scores indicate higher levels of CU traits. Previous studies have demonstrated acceptable reliability and validity of the ICU in Chinese youth [42]. In the current study, the Cronbach’s α coefficient for the total score was 0.77.

#### State-trait anxiety inventory-trait version (STAI-T)

The STAI-T is a commonly used measure to assess trait anxiety with 20 items. Each item is measured on a 4-point Likert scale ranging from 0 (does not apply at all) to 4 (does not apply at all). Higher scores indicate higher levels of trait anxiety. The Chinese version of this scale had shown acceptable reliability and validity [43]. In the current study, the Cronbach’s α coefficient for the total score was 0.81.

#### The reactive–proactive aggression questionnaire (RPQ)

The RPQ is a self-report questionnaire to measure aggressive behaviors in youth [44]. This scale consists of two subscales with 23 items, including 12 items of proactive aggression and 11 items of reactive aggression. Each item is measured on a 3-point Likert ranging from 0 (never) to 2(often). Higher scores indicate higher levels of aggression. The Chinese version of the RPQ had shown acceptable psychometric properties in youth [45]. In the current study, the Cronbach’s α coefficient for the total score was 0.92.

#### Basic empathy scale (BES)

The BES is a 20-item scale to assess empathy in youth. It consists of two subscales, including Cognitive empathy (9 items) and Affective empathy (11 items). Each item is rated on a 5-point Likert ranging from 1 (does not apply at all) to 5 (does not apply at all). Higher scores indicate higher levels of basic empathy. It had acceptable reliability and validity in Chinese youth [46]. In the current study, the Cronbach’s α coefficient for the total score was 0.73.

### Procedure

The present study was approved by Human Subjects Review Committee at Guangzhou University and was obtained institutional authorization from the prison administrative bureau of Guangdong Province. To ensure voluntary participation and ethical standards, participants were informed about the nature of the research, their rights, and the procedures and informed consent was obtained from participants and their legal guardians prior to the investigation and permitted to request clarification about the questionnaire if they had doubts about any part of the questionnaires during the investigation. All participants voluntarily completed the paper-and-pencil questionnaires with the same order of measures in the classroom for 40–60 min under the supervision of psychology-trained graduate students. To enhance transparency, we have elaborated on the measures taken to maintain participant confidentiality and the procedures for secure data handling, including data anonymization, storage protocols, and any relevant safeguards implemented.

### Statistical analysis

Descriptive statistics and reliability coefficient analyses were conducted with SPSS [47]. The Confirmatory factor analysis (CFA) and latent profile analysis (LPA) were conducted with M*plus* 8.0 [48].

The CTQ item scores were used to conduct LPA to determine the distinct subgroups in the sample (*N* = 625). Several LPA models (ranging from 1-profile to 5-profiles) were evaluated using robust maximum likelihood (MLR). To prevent Local Likelihood Maxima, 200 random sets of beginning values and 50 final stage optimizations were utilized initially [48].

Several fit indices were evaluated in the latent profile models to identify the optimal model, including the lower Akaike Information Criterion (AIC), Bayesian Information Criterion (BIC), and the Sample-Size Adjusted Bayesian Information Criterion (SSA-BIC). Besides, the significance of Lo-Mendell-Rubin Test (LMR) and the Bootstrap Likelihood Ratio Test (BLRT) indicating the k-profile model was superior to the k-1 profile model. In addition, the Entropy value (ranging from 0 to 1) was if the entropy value is over 0.80, which indicates that the classification accuracy surpasses 90% [49]. Lastly, participants were classified into the profile with higher probability of membership. The model would be accepted with the average probability of all profiles is more than 0.80 [19].

After identifying the optimal model, the modified Bolck-Croom-Hagenaars (BCH) and Categorical distal outcome (DCAT) methods were used to examine the associations between childhood maltreatment subgroups and distal variables (i.e., psychopathic traits, callous-unemotional traits, anxiety, aggression and empathy).

## Results

Descriptive statistics and correlation matrix about childhood maltreatment and outcome variables in Chinese juvenile offenders are presented in Table 1. The childhood maltreatment had moderately positive relationships with psychopathy, CU traits, anxiety and aggression, while it had a negative association with empathy.

**Table 1:** Pearson correlations, means, and standard deviations for main variables (*N* = 625)

	CTQ	APSD	PSCD	YPI	ICU	STAI-T	RPQ	BES
CTQ								
APSD	0.342**							
PSCD	0.295**	0.625**						
YPI	0.331**	0.670**	0.765**					
ICU	0.324**	0.536**	0.357**	0.445**				
STAI-T	0.350**	0.465**	0.215**	0.335**	0.439**			
RPQ	0.253**	0.613**	0.661**	0.704**	0.410**	0.386**		
BES	-0.119**	-0.189**	-0.125**	-0.217**	-0.441**	-0.065	-0.061	
*M* (*SD*)	52.69(9.75)	10.82(4.93)	16.34(7.35)	88.24(18.40)	50.01(8.32)	44.61(8.06)	11.77(9.04)	68.23(8.74)

*Notes.* *p <.05, **p <.01. CTQ = Childhood Trauma Questionnaire, APSD = Antisocial Process Screening Device, PSCD = Proposed Specifiers for Conduct Disorder Scale, YPI = Youth Psychopathic Traits Inventory, ICU = Inventory of Callous-Unemotional, STAI-T = State-Trait Anxiety Inventory-Trait version, RPQ = Reactive–Proactive Aggression Questionnaire, BES = Basic Empathy Scale. M = Mean, SD = standard deviations

Table 2 shows the LPA model fit indices from the one- to five-profiles model in the sample (*N* = 625). According to the results of the AIC, BIC, SSA-BIC and Entropy, the four-profile solution performed slightly better. The *p* value of LMR test was not significant when comparing the three-profile and four-profile model (*p* >.05; see Table 2). The three-profile model might be the better solution [50]. Nonetheless, the proportion of the three-profile model showed insufficient sample sizes (4.3%, *N* = 26). The two-profile model performs better than the three-profile model according to the value of the entropy. Based on parsimony and interpretability, the two-profile solution was selected as the optimal model.

**Table 2:** Model fit indices of the latent profile analysis in chinese juvenile offenders

Model	Log-likelihood	N. of free parameters	AIC	BIC	SSA-BIC	Entropy	LMR p	BLRT p
1	-3811.204	12	7646.407	7699.660	7661.562	-	-	-
**2**	**-3457.130**	**19**	**6952.260**	**7036.577**	**6976.255**	**0.890**	**< 0.001**	**< 0.001**
3	-3296.904	26	6645.808	6761.189	6678.643	0.868	< 0.001	< 0.001
4	-3208.902	33	6483.803	6630.249	6525.479	0.870	0.125	< 0.001
5	-3159.097	40	6398.194	6575.704	6448.709	0.876	0.244	< 0.001

*Notes.* AIC = Akaike Information Criteria; BIC = Bayesian Information Criteria; SSA-BIC = Sample-Size Adjusted BIC; LMR *p* = *p* value of the Lo-Mendell-Rubin test; BLRT *p* = *p* value of the Bootstrap Likelihood Ratio Test

**Fig. 1:**
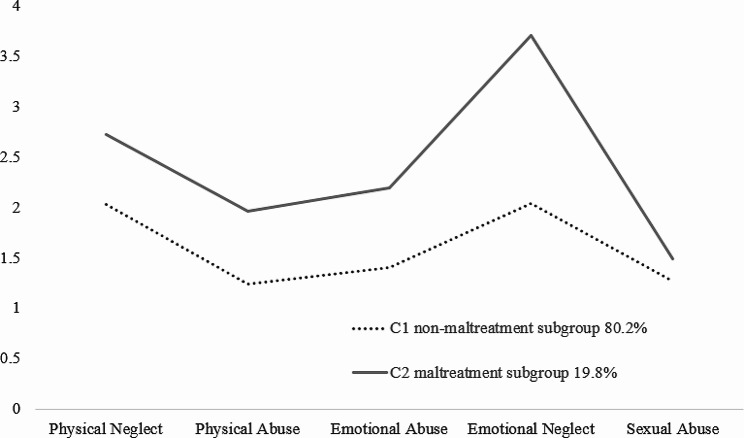
Latent profiles two subgroups of childhood maltreatment *Notes.* Class 1 = non-maltreatment subgroup; Class 2 = maltreatment subgroup

The means and standard errors of the maltreatment measures for each profile were reported in Table 3. Also, Fig. 1 shows the two maltreatment profiles based on the subscales of the CTQ-SF. Class 1 (80.2% of participants) showed had lower scores on all dimensions and therefore was labelled as “non-maltreatment subgroup” and class 2 (19.8% of participants) was characterized by high scores on all factors and labelled as “maltreatment subgroup”. Class 2 scored particularly higher in the emotional neglect compared with other dimensions. Both classes had a lowest score in sexual abuse.

**Table 3:** The profile membership of childhood maltreatment and scores for five indicators

	*N*	%	Latent profile^a^		CTQ-PN	CTQ-PA	CTQ-EA	CTQ-EN	CTQ-SA
			Class 1	Class 2					
Class 1	170	0.27	0.976		2.03(0.03)	1.25(0.02)	1.40(0.02)	2.04(0.05)	1.27(0.02)
Class 2	490	0.69		0.953	2.72(0.07)	1.96(0.14)	2.20(0.12)	3.70(0.10)	1.49(0.07)

*Notes.* Class 1 = non-maltreatment subgroup; Class 2 = maltreatment subgroup. Information for CTQ descriptive statistics is presented as M(SD). CTQ = Childhood Trauma Questionnaire; EA = emotional abuse; PA = physical abuse; SA = sexual abuse; EN = emotional neglect; PN = physical neglect

^a^ Average probabilities of profile membership

Table 4 shows the results of chi-square tests using modified BCH and DCAT between two latent profiles and outcome variables. As seen in Table 4, the two profiles had significant differences on psychopathy and other outcome variables (*p* <.05). Maltreatment subgroup had significantly greater values of the total scores of the APSD, PSCD, YPI, ICU, STAI and RPQ while it had a lower value of the BES.

**Table 4:** Mean differences in outcome variables across profiles (*N* = 625)

Variable	Class 1(*N* = 507)	Class 2(*N* = 118)	BCH χ^2^
APSD	10.12(0.21)	13.66(0.52)	36.55**
PSCD	15.79(0.33)	18.58(0.77)	10.51*
YPI	86.54(0.77)	95.15(2.21)	12.85**
ICU	48.66(0.35)	55.49(0.85)	51.72**
STAI-T	43.50(0.35)	49.11(0.79)	40.12**
RPQ	11.06(0.39)	11.68(1.00)	10.81*
BES	68.69(0.39)	66.38(0.86)	5.61*

*Notes.* *p <.05, **p <.01. Class 1 = non-maltreatment subgroup; Class 2 = maltreatment subgroup. CTQ = Childhood Trauma Questionnaire, APSD = Antisocial Process Screening Device, PSCD = Proposed Specifiers for Conduct Disorder Scale, YPI = Youth Psychopathic Traits Inventory, ICU = Inventory of Callous-Unemotional, STAI-T = State-Trait Anxiety Inventory-Trait version, RPQ = Reactive-Proactive Aggression Questionnaire, BES = Basic Empathy Scale. The standard error (SE) are also presented

## Discussion

In this study, we explored the profiles of childhood maltreatment in a sample of Chinese juvenile offenders using latent profile analysis. We identified two meaningful profiles in Chinese juvenile offenders, including non-maltreatment subgroup and maltreatment subgroup. Also, there were significant association between two profiles and outcome variables (i.e., psychopathy, CU traits, aggression, anxiety, empathy).

### Childhood maltreatment profiles

Two profiles were identified in this sample of Chinese juvenile. One profile was characterized by no or low probabilities of experiencing maltreatment. Most participants were classified into this profiles with a large proportion of 80%. It was consistent with a previous systematic review [51], which showed that most of the studies covered the low/no/mild maltreatment subgroup with the largest profile proportion [28]. For example, Aebi et al. (2015) identified no/mild maltreatment subgroup (76%) in detained male adolescent offenders from Austria [28]. Zhang and colleagues (2022) reported that 89% of the adolescents identified in the “no maltreatment” profile [22]. These findings supported majority of youth reported none or minimal endorsement of maltreatment types.

Compared with non-maltreatment subgroup, maltreatment subgroup was characterized by a greater level of all types of maltreatment, which indicated that this profile showed the co-occurrence of all types of maltreatment and participants were classified into this class with 20%. These distinct subgroups were consistent with the previous findings of maltreatment profiles based on the severity of each type [22, 25, 52]. For example, Romano and colleagues [25] reported that two profiles were found with 79% low/no abuse and 21% physical, sexual, emotional, and emotional neglect (21%). According to these findings from person-centered analyses, the juvenile offenders who experienced one type of maltreatment were more likely to experience other type of maltreatment, which was in line with findings from earlier studies [27]. Among those who reported a history of childhood maltreatment, 60% reported more than one type of maltreatment [52]. Similarly, most juvenile offenders also reported a history of more than one traumatic experience [11]. It demonstrated that juvenile youth with one type of maltreatment may have escalated risks for other types.

Besides, we found there were some similarities in both profiles although these two profiles had distinct differences from the severity of maltreatment. For example, the sexual abuse was both low in two profiles. One possible explanation might be the lower reported prevalence in Chinese samples. Prior studies found that sexual abuse experiences in collectivist cultures with lower rates of reported prevalence than in individualistic cultures. In the values of Asian culture, sexuality is often regarded as taboo, and the abusers shame themselves talking about the sexual abuse experience [53]. This may possibly reduce the prevalence of sexual abuse by self-rating among Chinese youth prisoners.

In addition, two profiles were characterized in higher levels of physical neglect and emotional neglect and lower levels in others dimensions described in the abuse. This pattern highlighted the feature of the childhood maltreatment in this sample, which indicated that neglect was the main feature. This was consistent with the previous findings of physical and emotional neglect as the most common form of childhood maltreatment among Chinese children and adolescents [54]. Such findings supported that neglect had a greater prevalence in Chinese youth. One possible reason was associated with the characteristics of participants. A meta-analytical finding suggested that neglect had a strong association with left-behind children [54]. Prolonged separation from parents significantly increases the likelihood of youth experiencing neglect.

Compared with physical neglect, emotional neglect was particularly at a higher level in the maltreatment subgroup. This finding suggested that this profile was remarkably characterized by emotional neglect, which was in line with the largest proportion of emotional neglect in a large Chinese children and juveniles [54]. Also, the highlight of emotional neglect may support previous research into this feature as an indirect effect on juvenile violent delinquency in Chinese juvenile offenders [55].

### Childhood maltreatment profiles and psychopathy

Maltreatment subgroup showed a significantly higher level of psychopathy. Compared with non-maltreatment subgroup in Chinese juvenile offenders across multiple self-report measures. These were consistent with previous meta-analytical findings of the associations between maltreatment and psychopathy from variable-centered approach [30]. The current findings supported that there was a link between maltreatment profiles and overall psychopathy in Chinese juvenile offenders from both variable- and person-centered approach and across self-report measures including the APSD, the PSCD and the YPI. It is suggested that the association between maltreatment profiles and psychopathy can be generalized to Chinese juvenile offenders, and this connection is further substantiated through the use of multiple self-report measures.

### Childhood maltreatment profiles and callous-unemotional traits

For the affective facet of psychopathy, maltreatment subgroup with particular higher emotional neglect had also greater level of CU traits and lower empathy. This was in accordance with the findings of maltreatment and CU traits in the previous studies [33, 56]. Emotional neglect is one type of childhood maltreatment in which the affection need of individuals are consistently neglected. For example, emotional neglect positively predicted CU traits among incarcerated boys in the US [56]. Furthermore, this also provide supports for the findings of CU traits as a mediator for the associations between childhood maltreatment and violent delinquency in Chinese juvenile offenders [55]. In summary, the present finding extended the understanding the associations between maltreatment and CU traits. Poor emotional experiences by neglectful surroundings may raise the risks of the development of CU traits and later delinquency.

### Childhood maltreatment profiles and aggression and anxiety

Furthermore, maltreatment subgroups also had higher level of aggression and anxiety. This supported the previous findings of multiple maltreatment associated with externalizing behaviors including aggressive behaviors and internalizing behaviors including anxiety in male adolescent offenders [20, 28]. Also, such findings were consistent with the findings of severe-maltreated profiles with higher aggression and anxiety in both Chinese and UK children [26]. These suggested that multiple maltreatment subgroups displayed higher aggression and anxiety in both Western and non-Western samples. Aggression and anxiety have been considered as a particularly common externalizing and internalizing problems. The finding of the association between multiple maltreatment and both aggression and anxiety may demonstrate that childhood maltreatment with combined types possibly raise the greater risk of both externalizing and internalizing consequences.

### Implications

The findings of the current study might provide further implications for understanding the maltreatment profiles and their associations with multiple consequences and clinical practice in policy-making and forensic context. First, this study suggested that two patterns of childhood maltreatment were uncovered (non-maltreatment subgroup and maltreatment subgroup) using latent profile analysis. Maltreatment subgroup displayed the combination of multiple maltreatment especially emotional neglect. For the clinical implications, this may encourage the measures informing on multiple experience in assessing maltreatment. We recommend employing instruments that capture experiences of various forms of abuse. The assessment of child abuse and maltreatment through standardized self-report measures has already been endorsed by the official guidelines of the American Psychiatric Association [57].

Second, examining a history of maltreatment can contribute to explaining the development of offending behavior in adolescents and aid in the risk assessment for subsequent delinquency [58]. Multiple experience of maltreatment may raise the greater risks of psychopathic personality including callous unemotional traits and lack of empathy, aggression, anxiety. These suggested that the combination of multiple maltreatment is the optimal choice for identifying juvenile offenders at the risk of developing psychopathy, aggression, anxiety. Therefore, it may focus on those juvenile offenders with multiple maltreatment in the intervention. Third, the association between maltreatment and psychopathy remained stable across different self-report measures. The self-report instruments were reliable for understanding the connections between maltreatment and psychopathy.

### Limitations and future directions

Several limitations might be noted in the present study. First, the participants were selected from Chinese juvenile offenders in China. Therefore, it was unclear whether the findings would be generalized to other samples with different characterizes (e.g., culture). Future studies may consider the comparison on the maltreatment profiles and psychopathy across culture. Second, the present study used a self-report rating to assess variables. The data may exhibit response bias influenced by individuals’ introspective abilities. The willingness to disclose adverse experiences, especially within offender samples, becomes crucial, particularly when addressing sensitive topics like childhood maltreatment. Future studies may consider combine with multiple ratings (e.g., experiment, clinical interview). Third, this study employed a cross-sectional design and therefore it may not be able to discover a causal relationship between childhood maltreatment and psychopathy. The longitudinal study needs to be carried out to examine the causal relationship.

## Data Availability

The datasets are available from the corresponding author upon reasonable request.

## Declarations

### Institutional approval

The present study was approved by Human Subjects Review Committee at Guangzhou University and was obtained institutional authorization from the prison administrative bureau of Guangdong Province.
